# Antibacterial and EGFR-Tyrosine Kinase Inhibitory Activities of Polyhydroxylated Xanthones from *Garcinia succifolia*

**DOI:** 10.3390/molecules191219923

**Published:** 2014-11-28

**Authors:** Susawat Duangsrisai, Kiattawee Choowongkomon, Lucinda J. Bessa, Paulo M. Costa, Nurmuhammat Amat, Anake Kijjoa

**Affiliations:** 1 *ICBAS*—Instituto de Ciências Biomédicas Abel Salazar—Universidade do Porto, Rua de Jorge Viterbo Ferreira 228, 4050-313 Porto, Portugal; E-Mails: fscissw@ku.ac.th (S.D.); lbessa@ciimar.up.pt (L.J.B.); pmcosta@icbas.up.pt (P.M.C.); 2 Department of Botany, Faculty of Science, Kasetsart University, Paholyothin Road, Chatuchuck, Bangkok 10900, Thailand; 3 Department of Biochemistry, Faculty of Science, Kasetsart University, Paholyothin Road, Chatuchuck, Bangkok 10900, Thailand; E-Mail: kiattawee@hotmail.com; 4 Interdisciplinary Centre of Marine and Environmental Research (CIIMAR), Universidade do Porto, Rua dos Bragas 289, 4050-123 Porto, Portugal; 5 Traditional Uighur Medicine Department, Xinjiang Medical University, 393 Medical University Road, Urumqi 830011, Xinjiang, China; E-Mail: nur818@hotmail.com

**Keywords:** antibacterial activity, antibiotic synergism, EGFR-tyrosine kinase inhibitory activity, *Garcinia succifolia*, multidrug-resistant bacteria, polyhydroxylated xanthone

## Abstract

Chemical investigation of the methanol extract of the wood of *Garcinia succifolia* Kurz (Clusiaceae) led to the isolation of 1,5-dihydroxyxanthone (**1**), 1,7-dihydroxyxanthone (**2**), 1,3,7-trihydroxyxanthone (**3**), 1,5,6-trihydroxyxanthone (**4**), 1,6,7-trihydroxyxanthone (**5**), and 1,3,6,7-tetrahydroxyxanthone (**6**). All of the isolated xanthones were evaluated for their antibacterial activity against bacterial reference strains, two Gram-positive (*Staphylococcus aureus* ATTC 25923, *Bacillus subtillis* ATCC 6633) and two Gram-negative (*Escherichia coli* ATCC 25922 and *Pseudomonas aeruginosa* ATCC 27853), and environmental drug-resistant isolates (*S. aureus* B1, *Enteroccoccus faecalis* W1, and *E. coli* G1), as well as for their epidermal growth factor receptor (EGFR) of tyrosine kinase inhibitory activity. Only 1,5,6-trihydroxy-(**4**), 1,6,7-trihydroxy-(**5**), and 1,3,6,7-tetrahydroxyxanthones (**6**) exhibited antibacterial activity against Gram-positive bacteria, however none was active against vancomycin-resistant *E. faecalis*. Additionally, 1,7-dihydroxyxanthone (**2**) showed synergism with oxacillin, but not with ampicillin. On the other hand, only 1,5-dihydroxyxanthone (**1**) and 1,7-dihydroxyxanthone (**2**) were found to exhibit the EGFR-tyrosine kinase inhibitory activity, with IC_50_ values of 90.34 and 223 nM, respectively.

## 1. Introduction

**Figure 1:**
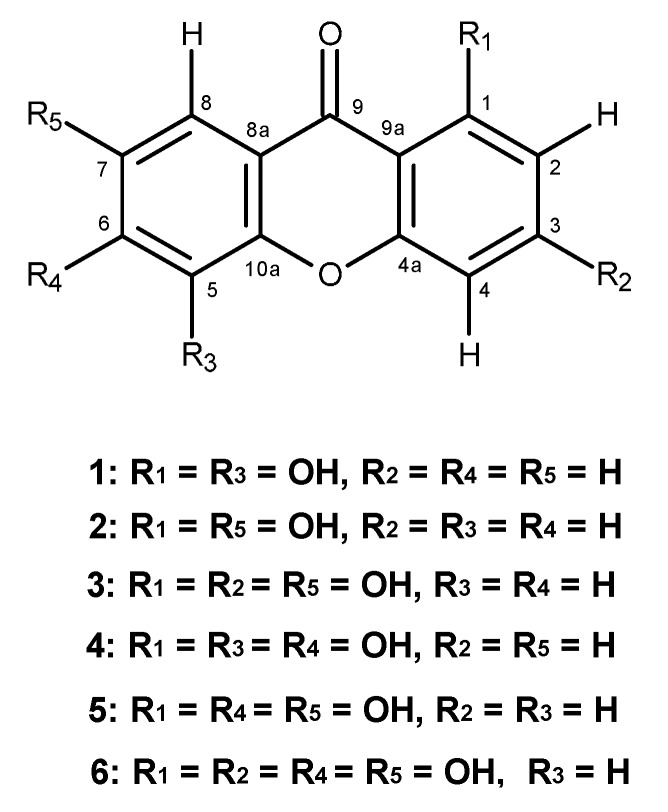
Polyhydroxylated xanthones from *G. succifolia* Kurz.

Many plants of the genus *Garcinia* (Family Clusiaceae) have been used in traditional medicine in several parts of the world for treatment of the most different illnesses [1]. In Thailand, where twenty-nine species have been observed [2], *G. mangostana*, *G. speciosa* and *G. cowa* have been widely used in Thai folk medicine. For example, the fruit hull of *G. mangostana* is used for healing of skin infections and wounds [3], whereas the bark and heartwood of *G. speciosa* are used for treatment of blood disorders, and its pericarp is used for the treatment of diarrhea [4]. Additionally, the bark, latex and root of *G. cowa* are used as an antifever agent while its fruits and leaves are used for indigestion, improvement of blood circulation and also as an expectorant [5,6]. The plants of this genus are also a rich source of xanthones [7], many of which exhibit interesting biological and pharmacological activities such as antibacterial [8,9] and anticancer properties [10]. As part of our ongoing research on the search for anticancer agents and antibiotics from natural sources, and since there are no previous reports on the chemical components and biological activity of *G. succifolia*, we have investigated its chemical constituents to evaluate their antibacterial activity against bacterial reference strains and multidrug-resistant isolates, as well as their inhibitory activity against the tyrosine kinase of epidermal growth factor receptor (EGFR). Examination of the methanol crude extract of the wood of *G. succifolia* Kurz, collected from Northern Thailand, led to the isolation of six polyhydroxylated xanthones: 1,5-dihydroxyxanthone (**1**), 1,7-dihydroxyxanthone (**2**), 1,3,7-trihydroxyxanthone (**3**), 1,5,6-trihydroxyxanthone (**4**), 1,6,7-trihydroxyxanthone (**5**), and 1,3,6,7-tetra-hydroxyxanthone (**6**) (Figure 1). All the xanthones were tested against bacterial reference strains: Gram-positive (*Staphylococcus aureus* ATCC 25923 and *Bacillus subtillis* ATCC 6633) and Gram-negative (*Escherichia coli* ATCC 25922 and *Pseudomonas aeruginosa* ATCC 27853), *Candida albicans* ATCC 10231, as well as against multidrug-resistant bacteria isolated from the environment. Furthermore, the potential synergism between these xanthones and antibiotics was also evaluated against multidrug-resistant bacteria: methicillin-resistant *S. aureus* (MRSA) and vancomycin-resistant enterococci (VRE). Xanthones **1**–**6** were also tested for their inhibitory activity against the EGFR protein tyrosine kinase, an important protein target for antitumor drug discovery.

## 2. Results and Discussion

In the past 30 years, treatment of infections caused by Gram-positive bacteria has been more problematic than ever, with infections being caused by multidrug-resistant organisms, particularly methicillin-resistant staphylococci, penicillin- and erythromycin-resistant pneumococci, and vancomycin-resistant enterococci [11]. Although there is a continuing effort in the pharmaceutical industry to develop new antimicrobial agents for the treatment of resistant infections, pursuing new antibiotic drugs is still an important strategy to combat the multidrug-resistant bacteria that are spreading both in the community and clinical setting [12]. In our pursuit for antibiotics of natural products origin [13], we have investigated antibacterial activity of six polyhydroxylated xanthones: 1,5-dihydroxyxanthone (**1**) [14], 1,7-dihydroxyxanthone (**2**) [15], 1,3,7-trihydroxyxanthone (**3**) [14], 1,5,6-trihydroxyxanthone (**4**) [16], 1,6,7-trihydroxyxanthone (**5**) [17] and 1,3,6,7-tetrahydroxy-xanthone (**6**) [18], isolated from a Thai collection of *G. succifolia* Kurz, and whose structures were established based on the ^1^H-, ^13^C-NMR and the HRMS data, as well as by comparison of their NMR data with those previously reported, against two Gram-positive (*S. aureus* ATCC 25923 and *B. subtilis* ATCC 6633), two Gram-negative (*E. coli* ATCC 25922 and *P. aeruginosa* ATCC 27853), and multidrug-resistant bacteria isolated from the environment, *S. aureus* B1 (isolated from a public bus), *E. faecalis* W1 (isolated from river water) and *E. coli* G1 (isolated from seagull feces), as well as against *C. albicans* ATCC 10231. The results (Table 1) show that 1,5,6-trihydroxyxanthone (**4**) and 1,6,7-trihydroxyxanthone (**5**) presented significant MIC values against Gram-positive bacteria. 1,5,6-Trihydroxyxanthone (**4**) showed a MIC value of 64 µg/mL against *S. aureus* ATCC25923 and *B. subtilis* ATCC 6633, while 1,6,7-trihydroxyxanthone (**5**) showed the MIC values of 64 and 128 µg/mL, respectively, against the same reference strains. On the contrary, 1,3,6,7-tetrahydroxy-xanthone (**6**) exhibited weaker activity, showing a MIC value of 256 µg/mL against both reference strains. Interestingly, only 1,5,6-trihydroxyxanthone (**4**) and 1,6,7-trihydroxyxanthone (**5**) were active against *S. aureus* MRSA, showing a MIC value at 64 µg/mL. However, none of the xanthones tested was active against vancomycin-resistant *E. faecalis.* Additionally, 1,5,6-trihydroxyxanthone (**4**) and 1,6,7-trihydroxyxanthone (**5**) also inhibited growth of *C. albicans* at a concentration of 256 µg/mL.

**Table 1:** Antimicrobial activity (MIC, MBC and MFC) against several bacterial strains (reference and multidrug-resistant strains) and a reference fungal strain (*C. albicans*).

	*S. aureus* ATCC 25923		*B. subtilis* ATCC 6633		*P. aeruginosa* ATCC 27853		*E. coli* ATCC 25922		*S. aureus* B1 (MRSA)		*E. faecalis* W1 (VRE)		*C. albicans* ATCC 10231	
**Xanthones**	MIC	MBC	MIC	MBC	MIC	MBC	MIC	MBC	MIC	MBC	MIC	MBC	MIC	MFC
**1**	˃256	-	˃256	-	˃256	-	˃256	-	˃256	-	˃256	-	˃256	-
**2**	˃256	-	˃256	-	˃256	-	˃256	-	˃256	-	˃256	-	˃256	-
**3**	˃256	-	˃256	-	˃256	-	˃256	-	˃256	-	˃256	-	˃256	-
**4**	64	˃256	64	˃256	˃256	-	˃256	-	64	˃256	˃256	-	256	256
**5**	64	˃256	128	˃256	˃256	-	˃256	-	64	˃256	˃256	-	256	˃256
**6**	256	˃256	256	˃256	˃256	-	˃256	-	˃256	-	˃256	-	˃256	-

(-): Not determined. MIC: minimum inhibitory concentration; MBC: minimum bactericidal concentration; MFC: minimum fungicidal concentration.

**Table 2:** Combined effect of antibiotics and polyhydroxylated xanthones (15 µg/disc) against three multidrug-resistant isolates, using the disc diffusion method (inhibition halos are expressed in mm).

	*E. coli* G1					*S. aureus* B1 (MRSA)				*E. faecalis* W1 (VRE)			
	Antibiotics												
**Xanthones**	Na	CIP	AMP	CTX	S	Na	OX	AMP	CIP	Na	VA	AMP	E
**1**	8	8	8	12	8	-	-	-	-	-	-	21	9
**2**	9	9	9	12	8	9	**12**	**11**	9	8	**12**	21	9
**3**	7.5	7.5	7.5	12	7.5	8	8	8	8	-	9	21	11
**4**	7.5	7.5	7.5	12	7.5	-	-	-	-	-	10	21	12
**5**	7.5	7.5	7.5	12	7.5	-	-	7.5	-	-	9	21	11
**6**	7.5	7.5	7.5	12	7.5	-	-	7.5	-		9	21	11
**Control**	-	-	12	-	-	-	-	-	-		9	21	11

Control: Antibiotic with no xanthone; Na: No antibiotic; CIP: Ciprofloxacin; AMP: Amplicillin; CTX: Cefotaxime; S: Streptomycin; OX: Oxacillin; VA: Vancomycin; E: Erythromycin. (-): indicates no halo of inhibition.

In order to verify if any of the xanthones tested was able to exert a synergistic effect with antibiotics against multidrug-resistant strains, a disc diffusion method was performed. The results (Table 2) show that only 1, 7-dihydroxyxanthone (**2**) showed potential synergy with oxacillin (OX) and ampicillin (AMP) against MRSA and with vancomycin against VRE. Consequently, the Checkerboard assay was carried out and the results (Table 3), represented by the FIC index, confirmed the synergistic effect (FIC index < 0.312) of a combination of 1,7-dihydroxyxanthone (**2**) and oxacillin (OX), while there was no synergistic effect observed for the combination of 1,7-dihydroxyxanthone (**2**) and ampicillin (AMP) (FIC index ˃ 0.5) against the MRSA isolate or with vancomycin (VA) (FIC index > 1) against VRE isolate.

**Table 3:** MIC value of 1,7-dihydroxyxanthone (**2**) in combination with oxacillin (OX), amplicillin (AMP) or Vancomycin (VA), and respective fractional inhibitory concentration (FIC) index obtained against a MRSA (*S. aureus* B1) or VRE (*E. faecalis* W1) by a checkerboard method.

	MIC (µg/mL)				
	**2** alone	**OX** alone	**2** with **OX**	**OX** with **2**	**ΣFIC**
*S. aureus* **B1**	˃256	128	16	32	˂0.312 *
	**2** alone	**AMP** alone	**2** with **AMP**	**AMP** with **2**	**ΣFIC**
*S. aureus* **B1**	˃256	128	128	64	0.5-1
	**2** alone	**VA** alone	**2** with **VA**	**VA** with **2**	**ΣFIC**
*E. faecalis* **W1**	˃256	256	˃256	256	>1

* FIC index—0.5 indicates synergy.

Although several prenylated xanthones from the Guttiferous plants have been reported for their antibacterial activity against methicillin-resistant *S. aureus* [8,9,19,20] and vancomycin-resistant enterococci [21], only few simple polyhydroxylated xanthones have been tested for antibacterial activity against multidrug-resistant bacteria and their synergistic effect with antibiotics.

Xanthones **1**–**6** were also tested for their capacity to inhibit the EGFR protein tyrosine kinase activity since EGFR has been found to involve several types of cancer such as ovary, prostrate, breast, lung, colon, head and neck cancer [22], and it is also the target of an expanding class of anticancer therapies such as gefitinib (Iressa^TM^, AstraZeneca, London, UK) [23] and erlotinib (OSI-774, Tarceva^TM^, OSI Pharmaceuticals, Northbrook, IL, USA) for lung cancer [24], and trastuzumab (Herceptin^TM^, Genentech, South San Francisco, CA, USA) for breast cancer [25]. The results (Table 4) show that, in the presence of 0.01 µM of 1,5-dihydroxyxanthone (**1**) and 1,7-dihydroxyxanthone (**2**), the relative activity of EGFR-tyrosine kinase was found to decrease to 19.01% and 51.31%, respectively, when compared to the control. In the presence of 1 µM of gefitinib, a standard EGFR-tyrosine kinase inhibitor, the activity of this enzyme was reduced to 32.65%. Thus, in comparison to gefitinib (IC_50_ = 33 nM) [26], 1,5-dihydroxyxanthone (**1**) (IC_50_ = 90.34 nM) and 1,7-dihydroxyxanthone (**2**) (IC_50_ = 223 nM) (Table 4) can be considered promising EGFR-tyrosine kinase inhibitors. Since xanthones **3**–**6** did not show significant inhibitory activity against the EGFR-tyrosine kinase, their IC_50_ were not determined. Examination of the structural features of xanthones **1**–**6** led to a suggestion that the EGFR prefers to bind the dihydroxyxanthones to the tri-and tetrahydroxy counterparts. Moreover, the positions of the hydroxyl groups also seem to play an important role in the activity of the polyhdroxylated xanthones since the hydroxyl groups on positions 3 and 6 were found to reduce their inhibitory activity, while the hydroxyl groups on positions 2 and 5 were found to increase it. To our knowledge, this is the first report on inhibitory activity of xanthones against the EGFR protein tyrosine kinase and consequently, dihydroxyxanthones can represent a new class of EGFR protein tyrosine kinase inhibitors.

**Table 4:** Thepercentage of relative activity of EGFR-tyrosine kinase in the presence of xanthones **1**–**6** (0.01 µM), and the IC_50_ values of **1** and **2**.

Compound	% Relative Activity *	IC_50_ (nM)
**1**	81.99 ± 3.30	90.34 ± 2.969
**2**	48.69 ± 9.34	223 ± 1.235
**3**	10.96 ± 9.34	-
**4**	no inhibition	-
**5**	13.71 ± 21.51	-
**6**	no inhibition	-
Gefitinib (standard inhibitor, 1 µM)	67.35 ± 2817	33
Control (no inhibitor)	0	-

* Standard deviations were calculated from triplicate experiments.

## 3. Experimental Section

### 3.1. General Experimentation Procedures

Melting points were determined on a Bock monoscope (Bibby Sterilin, Stratfordshire, UK) and are uncorrected. Infrared spectra were recorded on an ATI Mattson Genesis Series FTIR^TM^ (Madison, WI, USA) using WinFIRST Software. ^1^H and ^13^C-NMR spectra were recorded at ambient temperature on a Bruker AMC instrument operating at 300.13 and 75.4 MHz, respectively (Bruker Biosciences Corporation, Billerica, MA, USA). High resolution mass spectra were measured with a Waters Xevo QToF mass spectrometer coupled to a Waters Aquity UPLC system (Waters Corporations, Milford, MA, USA). Merck (Darmstadt, Germany) silica gel GF_254_ was used for preparative TLC, and a Merck Si gel 60 (0.2–0.5 mm) column was used for analytical chromatography.

### 3.2. Plant Material

*G. succifolia* Kurz (Clusiaceae) was collected at Huai Hong Khrai Royal Development Study Center, Doi Saket, Chiang Mai, Northern Thailand, in August 2007. The plant material was identified by Sombun Techapinyawat of the Department of Botany, Faculty of Science, Kasetsart University, Bangkok, Thailand, and the voucher specimen (herbarium n° 150687 BKF) is on deposit at the Office of Forest and Plant Conservation Research, National Park, Wildlife and Plant Conservation Department, 61 Phaholyothin Road, Chatuchak, Bangkok, Thailand.

### 3.3. Extraction and Isolation of Xanthones

Dried powdered wood (2.1 kg) was extracted with MeOH (3 × 8 L) at room temperature until exhaustion. The methanol solution was evaporated at reduced pressure to give a syrupy mass (150 g) of crude methanol extract, which was extracted with EtOAc (3 × 500 mL) with the aid of an ultrasound bath. The EtOAc extracts were combined and evaporated at reduced pressure to give a residue (49 g) which was dissolved in CHCl_3_ (200 mL), filtered with filter paper, and the CHCl_3_ solution was evaporated under reduced pressure to give crude chloroform extract (44 g) which was chromatographed over a 0.2–0.5 mm Merck Si gel column (320 g, 5 cm × 90 cm) and eluted with mixtures of CHCl_3_–petroleum ether and CHCl_3_–Me_2_CO, wherein 250 mL fractions were collected as follows: Frs 1–58 (CHCl_3_–petroleum ether, 7:3), 59–167 (CHCl_3_–petroleum ether, 9:1), 168–432 (CHCl_3_–Me_2_CO, 9:1), 433–565 (CHCl_3_–Me_2_CO, 7:3). Frs. 59–123 were combined (320 mg) and crystallized in a mixture of petroleum ether and CHCl_3_ to give 75.2 mg of 1,5-dihydroxyxanthone (**1**). Frs 171 (598.2 mg) was crystallized in a mixture of petroleum ether and CHCl_3_ to give 127.6 mg of 1,7-dihydroxyxanthone (**2**). The mother liquor (420 mg) was combined with frs 172–175 (620 mg), chromatographed over Si gel (47 g, 2.0 cm × 40 cm), and eluted with mixtures of petroleum ether–CHCl_3_, 100 mL fractions were collected as follows: sfrs 1–99 (petroleum ether–CHCl_3_, 3:7), 100–212 (petroleum ether–CHCl_3_, 1:9), 213–231 (CHCl_3_–Me_2_CO, 9:1). Sfrs 102–186 (238 mg) was recrystallized in a mixture of CHCl_3_ and Me_2_CO to give 64.7 mg of 1,7-dihydroxyxanthone (**2**). Purification of the mother liquor by TLC (silica gel, CHCl_3_–Me_2_CO–HCO_2_H, 9:1.0.1) gave an additional 12 mg of 1,7-dihydroxyxanthone (**2**). Frs 176–181 were combined (659.4 mg) and recrystallized in a mixture of CHCl_3_ and Me_2_CO to give 320 mg of 1,3,7-trihydroxyxanthone (**3**). Frs. 182–185 were combined (170 mg) and recrystallized from a mixture of CHCl_3_ and Me_2_CO to give 30.4 mg of 1,5,6-trihydroxy-xanthone (**4**). The mother liquor of frs 176–185 were combined and applied on a Si gel column (11 g, 1.5 cm × 13.5 cm), and eluted with mixtures of petroleum ether–CHCl_3_ and CHCl_3_–Me_2_CO, 100 mL sfrs were collected as follows: sfrs 1–40 (petroleum ether–CHCl_3_, 3:7), 41–51 (petroleum ether–CHCl_3_, 1:9), 52–65 (CHCl_3_–Me_2_CO, 9:1). Sfrs 15–21 were combined (14.3 mg) and purified by TLC (Si gel, CH_2_Cl_2_–Me_2_CO–HCO_2_H, 95:5:0.1) to give 1.4 mg of 1,7-dihydroxyxanthone (**2**). Sfrs 22–26 were combined (15 mg) and recrystallized in a mixture of CHCl_3_ and Me_2_CO to give 1.8 mg of 1,3,7-trihydroxyxanthone (**3**). Sfrs 27–65 were combined (75.8 mg) and purified by TLC (Si gel, CH_2_Cl_2_–Me_2_CO–HCO_2_H, 95:5:0.1) to give additional 12.3 mg of 1,5,6-trihydroxyxanthone (**4**). Frs. 186–204 were combined (782.1 mg) and chromatographed over Si gel (12 g, 1.5 cm × 13.5 cm), and eluted with mixtures of petroleum ether–CHCl_3_ and CHCl_3_–Me_2_CO, 100 mL fractions were collected as follows: sfrs 1–9 (petroleum ether–CHCl_3_, 3:7), 10–27 (petroleum ether–CHCl_3_, 1:9), 28–39 (CHCl_3_–Me_2_CO, 9:1). Sfrs 10–14 were combined and recrystallized in a mixture of CHCl_3_ and Me_2_CO to give 6.6 mg of 1,7-dihydroxyxanthone (**2**). Sfrs 15–27 were combined and recrystallized in a mixture of CHCl_3_ and Me_2_CO to give 6.4 mg of 1,5,6-trihydroxyxanthone (**4**). Frs 235–290 were combined (983 mg) and recrystallized in a mixture of CHCl_3_ and Me_2_O to give 288 mg of 1,3,6,7-tetrahydroxyxanthone (**6**), and the mother liquor was combined with frs 205–234, and subjected to column chromatography on Si gel (13.64 g, 1.5 cm × 13.5 cm) and eluted with mixtures of petroleum ether–CHCl_3_ and CHCl_3_–Me_2_CO, 100 mL fractions were collected as follows: sfrs 1–13 (petroleum ether–CHCl_3_, 3:7), 14–48 (petroleum ether–CHCl_3_, 1:9), 49–78 (CHCl_3_–Me_2_CO, 9:1). Sfrs 1–12 were combined (18.9 mg) and crystallized in a mixture of petroleum ether and CHCl_3_ to give 3.2 mg of 1,7-dihydroxyxanthone (**2**). Sfrs 25–30 were combined (16.8 mg) and recrystallized in mixture of CHCl_3_ and Me_2_CO to give 3.7 mg of 1,5,6-trihydroxyxanthone (**4**). Sfrs 31–48 were combined (52.2 mg) and recrystallized from a mixture of CHCl_3_ and Me_2_CO to give 21.9 mg of 1,6,7-trihydroxyxanthone (**5**). Frs. 291–385 were combined (2.14 g) and recrystallized in a mixture of CHCl_3_ and Me_2_CO to give 213.2 mg of 1,3,6,7-tetrahydroxyxanthone (**6**).

### 3.4. Antimicrobial Activity Bioassays

#### 3.4.1. Bacterial Strains

For the antimicrobial assays, xanthones **1**–**6** were tested against bacterial reference strains, two Gram-positive (*Staphylococcus aureus* ATCC 25923 and *Bacillus subtilis* ATCC 6633) and two Gram-negative (*Escherichia coli* ATCC 25922 and *Pseudomonas aeruginosa* ATCC 27853) bacteria; *Candida albicans* ATCC 10231; multidrug-resistant bacteria isolated from the environment, *S. aureus* B1 (isolated from a public bus), *Enterococcus faecalis* W1 (isolated from river water) and *E. coli* G1 (isolated from seagull feces). Bacteria were grown in Mueller-Hinton agar (MH–BioKar diagnostics, Allonne, France) from stock cultures, while *C. albicans* was grown in Sabouraud dextrose agar (SAB–BioKar Diagnostics, Allonne, France). MH and SAB plates were incubated at 37 °C prior to obtain fresh cultures for each *in vitro* bioassay.

#### 3.4.2. Determination of Minimum Inhibitory and Bactericidal/Fungicidal Concentrations

The minimum inhibitory concentrations (MIC) of xanthones **1**–**6** were determined using a broth microdilution technique, following the recommendations of the Clinical and Laboratory Standards Institute [27]. Stock solutions of 10 mg/mL (prepared by dissolving each xanthone in dimethylsulfoxide (DMSO-Applichem GmbH, Darmstadt, Germany) were serially diluted in Mueller-Hinton broth (MHB-BioKar Diagnostics, Allonne, France) to achieve in-test concentrations ranging from 2 to 256 µg/mL. Ciprofloxacin in the concentration range from 0.03125 to 16 μg/mL was used as control drug in the experiment. Each bacterial inoculum was prepared in MHB, while *C. albicans* inoculum was prepared in RPMI-1640 with l-glutamine, with MOPS and without NaHCO_3_ (Lonza, Walkersville, MD, USA). All inocula were standardized in order to obtain a concentration of 5 × 10^5^ CFU/mL in each inoculated well of the microtiter plate. The concentration of DMSO in the highest in-test concentration did not affect the microbial growth. The MIC was defined as the lowest concentration of xanthone that inhibited the visible growth. The minimum bactericidal/fungicidal concentration (MBC/MFC) was determined by spreading 10 µL on MH/SAB plates from the sample showing no visible growth, with further incubation for 24 h at 37 °C; the lowest concentration at which no growth occurred on MB/SAB plates was defined as the MBC/MFC, respectively. 

#### 3.4.3. Synergistic Activity Studies

##### Screening of Combined Effect between Xanthones and Antibiotics

A screening susceptibility test to assess the combined effect between the xanthones **1**–**6** and antibiotics was conducted using the disc diffusion method on MH, according to the procedure already described by Gomes *et al.* [13]. Briefly, multidrug-resistant isolates were picked from overnight cultures in MH, and suspensions were prepared in buffered peptone water (Oxoid, Basingstoke, England) by adjusting the turbidity to equal a 0.5 McFarland standard. A set of antibiotic discs (Oxoid) was selected based on the resistance of the isolates towards these antibiotics. Antibiotic discs alone (controls) and impregnated with 15 µL of a 1 mg/mL solution (in DMSO) of each xanthone were placed on the agar plate seeded with the respective bacteria. Fifteen microliter of DMSO impregnated in a sterile filter paper disc (6 mm in diameter) (Oxoid) was used as a negative control. Inoculated MH plates were incubated overnight at 37 °C. Each xanthone was tested in duplicate. Potential synergism was recorded when the halo of antibiotic discs impregnated with metabolites was greater than the halo of antibiotic discs or xanthone-impregnated disc alone. 

##### Synergy Test: Checkerboard Method

Based on the results of the previous assay, potential synergy between 1,7-dihydroxyxanthone (**2**) and oxacillin or ampicillin (Sigma-Aldrich, St. Louis, MO, USA) against MRSA (*S. aureus* B1), and vancomycin against VRE (*E. faecalis* W1) was checked using a broth microdilution checkerboard method, as already described [13]. Briefly, the stock solutions and serial two-fold dilutions of the tested xanthone and antibiotic to at least double the MIC were prepared according to the recommendations of CLSI [27]. The xanthone to be tested was serially diluted along the ordinate, while the antibiotic was diluted along the abscissa. A bacterial inoculum equal to a 0.5 McFarland turbidity standard was prepared in MHB. Each microtiter plate well was inoculated with 100 µL of a bacterial inoculum of 5 × 10^5^ CFU/mL, and the plates were incubated overnight at 37 °C. Two independent experiments in duplicate were performed. The fractional inhibitory concentration (FIC) was calculated as follows: FIC of drug A (FIC A) = MIC of drug A in combination/MIC of drug A alone, and FIC of drug B (FIC B) = MIC of drug B in combination/MIC of drug B alone. The FIC index (ΣFIC), calculated as the sum of each FIC, was interpreted as follows: ΣFIC ≤ 0.5, synergy; 0.5 < ΣFIC ≤ 4, no interaction; 4 < ΣFIC, antagonism [28].

### 3.5. Tyrosine Kinase Inhibition Assay

Xanthones **1**–**6** were dissolved in 100% dimethyl sulfoxide (DMSO) to a concentration of 35 µM, and used as stock solution. The working solution was prepared by diluting with 1× kinase buffer containing 2 mM DTT to concentration of 60 nM. Samples were kept at 4 °C prior to use. Tyrosine kinase inhibition assays by fluorescence were performed according to the manufacturer’s instruction. Briefly, 12.5 µL of kinase sample was incubated with 1.25 µL of the compound at room temperature for 5 min. Next, add 20 µL of Antibody Beacon detection complex plus substrate, and incubated at room temperature for 10 min in the dark. Subsequently, start the reaction by adding ATP with a final concentration of 0.5 mM/reaction, and the activities were determined using fluorescence microplate reader with excitation/emission at 485/535 nm.

### 3.6. Determination of IC_50_

Xanthones **1**–**6** were diluted in two-fold dilution (final concentration ranging from 0.975 nM to 500 nM), and 12.5 µL of the solution was added to 12.5 µL of the kinase sample, incubated at room temperature for 5 min. Next add 20 µL of Antibody Beacon detection complex plus substrate, and incubated at room temperature for 10 min. Subsequently, start the reaction by adding ATP with a final concentration of 0.5 mM/reaction. The activities were determined using fluorescence microplate reader with with excitation/emission at 485/535 nm. Non-linear regression dose response curves were plotted against activity and log inhibitor concentration. The fifty percent inhibitions (IC_50_) were calculated using the GraphPad Prism program (GraphPad Software Inc., San Diego, CA, USA).

## 4. Conclusions

The results obtained from this study revealed that polyhydroxylated xanthones are potential antibacterial agents, especially against the multidrug-resistant Gram-positive bacteria. Although, this study did not allow us to draw any conclusions about the structure activity relationship of the polyhydroxylated xanthones, it seems that the number and position of the hydroxyl groups on the xanthone nucleus can influence their antibacterial activity as well as their synergistic effect with antibiotics. With the emergence of multidrug-resistant organisms, combining natural products, such as xanthones, with antibiotics can be considered a useful approach to combat resistant bacteria. On the other hand, the capacity of the dihydroxyxanthones, namely 1,5-dihydroxyxanthone (**1**) and 1,7-dihydroxyxanthone (**2**) to inhibit the activity of EGFR protein tyrosine kinase, an important target of anticancer chemotherapies, can be considered as a support for polyhydroxylated xanthones as potential chemopreventive agents. However, further investigation of these xanthones on human cancer cell lines is necessary to support this hypothesis.

## References

[1] Kijjoa A., Vieira L.L.M., Brahmachari G. (2009). Triterpenes from the plants of the Family Clusiaceae (Guttiferae): Chemistry and Biological activities. Natural Products: Chemistry, Biochemistry and Pharmacology.

[2] Ritthiwigrom T., Laphookhieo S., Pyne S.G. (2013). Chemical constituents and biological activities of *Garcinia cowa* Roxb. Maejo Int. J. Sci. Technol..

[3] Paveen M., Khan N.U., Acchari B., Dutta P.K. (1991). A triterpene from *Garcinia mangostana*. Phytochemistry.

[4] Rukachaisirikul V., Pailee P., Hiranat A., Tuchinda P., Yoosook C., Kasisit J., Taylor W.C., Reutrakul V. (2003). Anti-HIV-1 protostane triterpenes and digeranylbenzophenone from trunk bark and stem of *Garcinia speciosa*. Planta Medica.

[5] Mahabusarakam W., Chairerk P., Taylor W.C. (2005). Xanthones from *Garcinia Cowa* Roxb. latex. Phytochemistry.

[6] Panthong K., Hutadilok-Towanata N., Panthong A. (2009). Cowaxanthone F, a new tetraoxygenated xanthone, and other anti-inflammatory and antioxidant compounds from *Garcina cowa*. Can. J. Chem..

[7] Kijjoa A., Gonzalez M.J., Pinto M., Nascimento M.S.J., Campos N., Mondranondra I.O., Silva A.M.S., Eaton G., Herz W. (2008). Cytotoxicity of Prenylated Xanthones and Other Constituents from the Wood of *Garcinia merguensis*. Planta Medica.

[8] Iinuma M., Tosa H., Tanaka T., Asai F., Kobayashi Y., Shimano R., Miyauchi K. (1996). Antibacterial activity of xanthones from Guttiferous plants against methicillin-resistant Staphylococcus aureus. J. Pharm. Pharmacol..

[9] Chomnawang M.T., Surrasmo S., Wongsariya K., Bunyapraphatsara N. (2009). Antibacterial activity of Thai medicinal plants against methicillin-resistant *Staphylococcus aureus*. Fitoterapia.

[10] Na Y. (2009). Recent cancer drug development with xanthone structures. J. Pharm. Pharmacol..

[11] Aksoy D.Y., Unal S. (2008). New antimicrobial agents for treatment of Gram-positive bacterial infections. Clin. Microbiol. Infect..

[12] Bassetti M., Merelli M., Temperoni C., Astilean A. (2013). New antibiotics for bad bugs: Where are we?. Ann. Clin. Microbiol. Antimicrob..

[13] Gomes N.M., Bessa L.J., Buttachon B., Costa P.M., Buaruang J., Dethoup T., Silva A.M.S., Kijjoa A. (2014). Antibacterial and antibiofilm activities of tryptoquivalines and meroditerpenes isolated from the marine-derived fungi *Neosartorya paulistensis*, *N. laciniosa*, *N. tsunodae*, and the soil fungi *N. fischeri* and *N. siamensis*. Mar. Drugs.

[14] Zhang Z., El Sohly H.N., Jacob M.R., Pasco D.S., Walker L.A., Clark A.M. (2002). Natural Products inhibiting *Candida albicans* secreted aspartic protease from *Tovomita krukovii*. Planta Medica.

[15] Kijjoa A., Gonzalez M.J., Afonso C.M., Pinto M.M.M., Anantachoke C., Herz W. (2000). Xanthones from *Calophyllum teysmannii* var. *inophylloide*. Phytochemistry.

[16] Wu Q.L., Wang S.P., Du L.J., Yang J.S., Xiao P.G. (1998). Xanthones from *Hypericum japonicum* and *H. henryi*. Phytochemistry.

[17] Jackson B., Locksley H.D., Scheinmann F. (1969). Extractives from Guttiferae. Part XIII. Isolation and structure of five xanthones from *Garcinia eugeniifolia* wall. J. Chem. Soc. C.

[18] Fu W.M., Zhang J.F., Wang H., Tan H.S., Wang W.M., Chen S.C., Zhu X., Chan T.M., Tse C.M., Leung K.S. (2012). Apoptosis induced by 1, 3, 6, 7-tetrahydroxyxanthone in hepatocellular carcinoma and proteomic analysis. Apoptosis.

[19] Dharmaratne H.R.W., Wijesinghe W.M.N.M., Thevanasem V. (1999). Antimicrobial activity of xanthones from *Calophyllum* species, against methicillin-resistant *Staphylococcus aureus* (MRSA). J. Ethnopharmacol..

[20] Rukachaisirikul V., Kamkaew M., Sukavisit D., Phongpaichit S., Sawangchote P., Taylor C.W. (2003). Antibacterial xanthones from the leaves of *Garcinia nigrolineata.*. J. Nat. Prod..

[21] Sakagami Y., Iinuma M., Piyasena K.G.N.P., Dharmaratne H.R.W. (2005). Antibacterial activity of α-mangostin against vancomycin resistant *Enterococci* (VRE) and synergism with antibiotics. Phytomedicine.

[22] Woodburn J.R. (1999). The epidermal growth factor receptor and its inhibition in cancer therapy. Pharmacol. Ther..

[23] Anderson N.G., Ahmad T., Chan K., Dobson R., Bundred N.J. (2001). ZD 1839 (Iressa), a novel epidermal growth factor receptor (EGFR) tyrosine kinase inhibitor, potently inhibits the growth of EGFR-positive cancer cell lines with or without erbB2 overexpression. Int. J. Cancer.

[24] Hidalgo M., Siu L.L., Nemunaitis J., Rizzo J., Hammond L.A., Takimoto C., Eckhardt S.G., Tolcher A., Britten C.D., Denis L. (2001). Phase I and pharmacology study of OSI-774, an epidermal growth factor receptor tyrosine kinase inhibitor in patients with advanced solid malignancies. J. Clin. Oncol..

[25] Harries M., Smith I. (2002). The development and clinical use of trastuzumab (Herceptin). Endocr. Relat. Cancer.

[26] Wakeling A.E., Guy S.P., Woodburn J.R., Ashton S.E., Curry B.I., Barker A.J., Gibson K.H. (2002). GEFITINIB (Gefitinib): An orally active inhibitor of epidermal growth factor signaling with potential for cancer chemotherapy. Cancer Res..

[27] Clinical and Laboratory Standards Institute (CLSI) (2011). Performance Standards for Antimicrobial Susceptibility Testing.

[28] Odds F.C. (2003). Synergy, antagonism, and what the chequerboard puts between them. J. Antimicrob. Chemother..

